# Remedy for Scrofulous Affections

**Published:** 1883-01

**Authors:** 


### BLACK WALNUT LEAVES AS A REMEDY IN SCROFULOUS AND CACHECTIC AFFECTIONS.

Dr. Orlando C. Farquhar, of Zanesville, O., brings to the notice of the profession the great medicinal value and curative properties possessed by the leaves of the common black walnut. He says: "I am personally cognizant of very many cures that an infusion of the leaves of the common black walnut has performed after the skil and prescriptions of many excellent physicians had signally failed. This remedy has never, to my knowledge, failed to cure or greatly benefit whenever used. That is saying a great deal for walnut leaves, and yet such is the truth; and 'facts and figures are stubborn things.' My father, Dr. E. A. Farquhar, Sr., has continuously used this remedy for the last quarter of a century. With a reputation and practice coextensive with the boundaries of the State, he had frequent opportunities for testing the virtues of the remedy As an alterative and depurative, possessing marked powers in the cure of all scrofulous and cachectic affections, its value has often been demonstrated. Syphylitic ulcerations and squamous diseases of the skin soon yield to the effects of an infusion of the leaves of the black walnut. Chronic ulcers of the indolent kind have been cured with this remedy, after every other remedy had been exhausted without benefit. Our plan has been to wash the parts affected, if external, two or three times daily with the infusion, and at the same time take it internally. In cases of syphylis, I often combine or add to the infusion an alcoholic solution of hydrargyrum corrosivum, with good results.

"M. Negrier, professor of the school at Angers in 1841, published an interesting memoir on the use of walnut leaves in scrofula, which he regards, after numerous experiments, as one of the best anti-scrofulous remedies that we possess. M. Negrier, as well as several other medical men at Angers, had long been in the habit of employing a decoction of walnut leaves as a lotion for scrofulous sores.

"M. Negrier concludes his memoir with the following directions for the preparations of walnut: 'The infusion is made with an ounce of leaves to twelve ounces of boiling water. It may be sweetened with sugar or the sirup presently to be noticed. Two or three cupfuls may be given daily, or even five. The decoction is made with a handful of the leaves boiled fifteen minutes in a quart of water. The extract may be made in the usual manner from the dried leaves. For the sirup eight grains of the extract are mixed with thirty-two scruples of common sirup; infants and young children may take two or three teaspoonfuls in the day; adults three drachms. The pills may be made of the extract, four grains in each; from two to four in the day.'"

